# Automated machine learning for endemic active tuberculosis prediction from multiplex serological data

**DOI:** 10.1038/s41598-021-97453-7

**Published:** 2021-09-09

**Authors:** Hooman H. Rashidi, Luke T. Dang, Samer Albahra, Resmi Ravindran, Imran H. Khan

**Affiliations:** Department of Pathology and Laboratory Medicine, University of California Davis, 4400 V Street, Sacramento, CA 95817 USA

**Keywords:** Tuberculosis, Machine learning, Diagnostic markers, Tuberculosis

## Abstract

Serological diagnosis of active tuberculosis (TB) is enhanced by detection of multiple antibodies due to variable immune responses among patients. Clinical interpretation of these complex datasets requires development of suitable algorithms, a time consuming and tedious undertaking addressed by the automated machine learning platform MILO (Machine Intelligence Learning Optimizer). MILO seamlessly integrates data processing, feature selection, model training, and model validation to simultaneously generate and evaluate thousands of models. These models were then further tested for generalizability on out-of-sample secondary and tertiary datasets. Out of 31 antigens evaluated, a 23-antigen model was the most robust on both the secondary dataset (TB vs healthy) and the tertiary dataset (TB vs COPD) with sensitivity of 90.5% and respective specificities of 100.0% and 74.6%. MILO represents a user-friendly, end-to-end solution for automated generation and deployment of optimized models, ideal for applications where rapid clinical implementation is critical such as emerging infectious diseases.

## Introduction

The etiologic agent of tuberculosis (TB), *Mycobacterium tuberculosis* (*M. tb.*), latently infects a third of the world’s population (approximately two billion) and leads to an estimated 10.4 million new cases of active disease (active TB) every year^1^. Active TB is responsible for 1.7 million deaths each year, making it the single largest cause of infectious disease deaths, ahead of HIV-AIDS (1.0 million deaths) and malaria (0.44 million deaths)^2^. Even more alarming, while deaths due to the other two leading infectious diseases (AIDS and malaria) are down trending, TB deaths continue to rise with no clear signs of reversal on the horizon^2^.

TB endemic countries face multiple challenges in treating this persistent infectious disease as suboptimal access to medical care makes both diagnosis and treatment difficult. However, a critical step towards effectively reducing this public health burden is improved, widespread, accurate, timely, and cost-effective testing, a task that powerful platforms such as multiplex microbead assay (MMIA) are well suited to. MMIA enables the simultaneous detection of antibodies and/or antigens efficiently for a wide range of infectious agents in host blood samples in a single reaction vessel. We have previously demonstrated the diagnostic validity of MMIA in adults with pulmonary tuberculosis based on testing for plasma antibodies to specific *M.tb* antigens in a TB endemic country, Pakistan^3–5^. The MMIA method inherently generates large volumes of data, therefore computational methods for analysis and interpretation of this data (although very time consuming) were an integral component of these studies^3,4^. While MMIA is a powerful method for accumulating large sets of immunologic data, our prior study demonstrated that optimal downstream analysis and interpretation of that data is equally important to transform these data into actionable and diagnostically reliable clinical results. Therefore, evaluation of a large set of diverse alternative algorithms using improved data mining approaches may further enhance this approach, enabling discovery of optimal classifiers that are capable of distinguishing TB from other mimickers and healthy subjects^6–8^.

In the last decade, researchers have improved methods for the development of high-throughput computational algorithms which extract biologically meaningful information from genomic and proteomic datasets whose increasingly complex and extensive nature challenges traditional methods^9,10^. Data mining techniques provide efficient and effective tools to observe and analyze large volumes of data by enabling elucidation of important patterns and correlations which may ultimately reveal the underlying mechanisms of biological function or disease^11–13^. Techniques within the artificial intelligence/machine learning and statistics realms paired with various visualization tools now allow the researcher to analyze and expose hidden information within data that can ultimately enhance predictive outcomes^9,11^.

**Figure 1:**
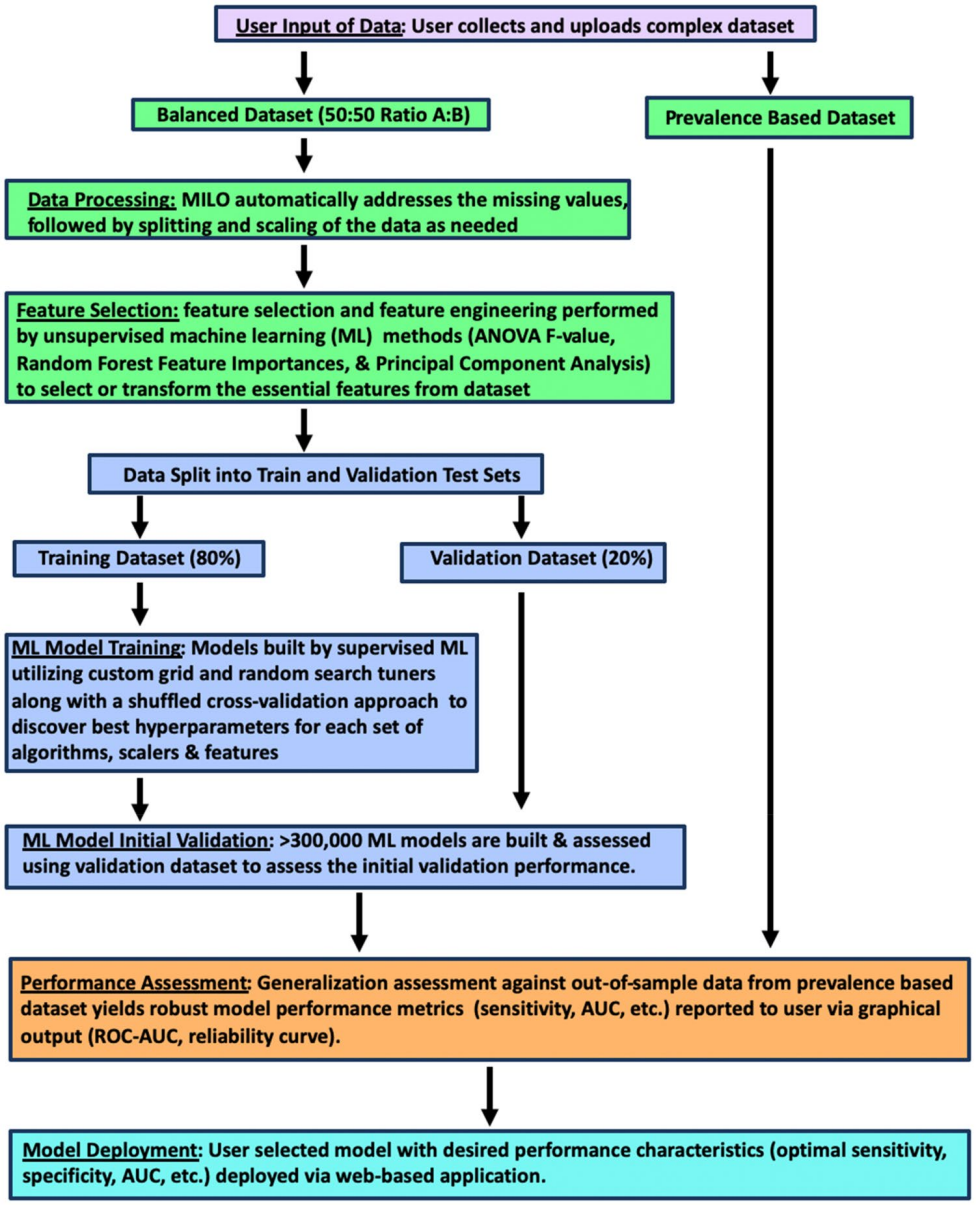
Schematic of the automated-machine learning platform MILO. Overview of the infrastructure and process for data processing, feature selection, and subsequent model training, building, initial validation, generalization testing and selection. MFI values for 31 anti-*M.tb.* antigens generated by multiplex microbead immunoassays comprise the balanced training dataset (Dataset A in this study). A large number of optimized models (> 300,000) were generated from the training dataset after data processing, feature selection, training, and validation. The true performance of the optimized models is then evaluated on the out-of-sample generalization (ideally prevalence-based) dataset (Datasets B and C in this study).

**Figure 2:**
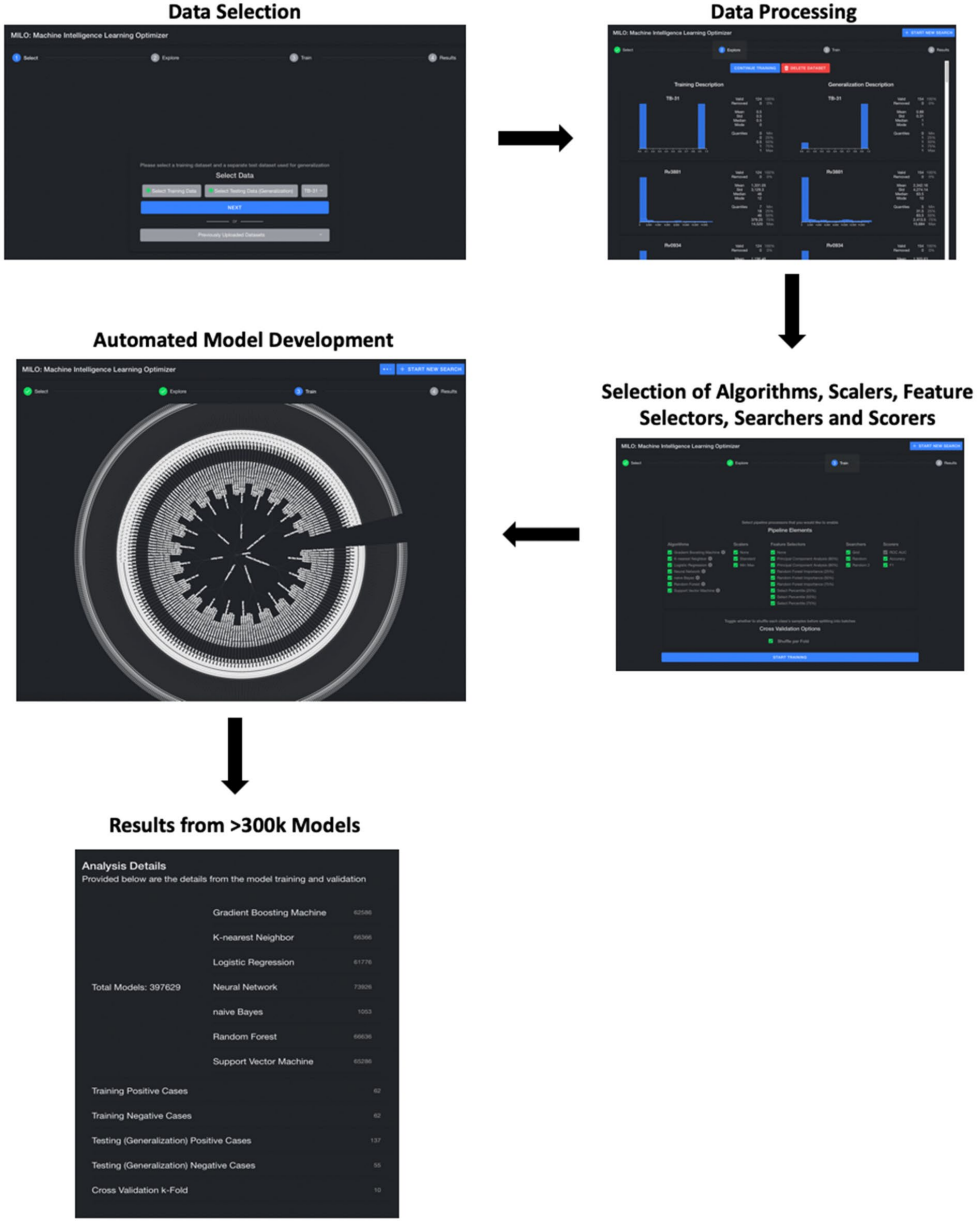
User interface for MILO. Stepwise overview of the user-friendly interface for the automated-machine learning platform MILO sequentially through the pipeline: data upload, data processing, selection of algorithms, scalers, feature selectors, searchers, and scorers, and assessment of model results from generalization testing.

The emergence of machine learning (ML) models in diagnostic medicine represent a thus far underutilized opportunity for extracting actionable information from existing data and hold great promise for improving patient care^6,14,15^. Recent studies have shown that ML models can improve diagnostic accuracy and clinical sensitivity/specificity in various disease entities^16,17^. Therefore, advancements in ML may help to bridge the gap in the diagnosis of tuberculosis and access to health care in TB endemic countries^18–20^. However, the use of ML in diagnostic medicine is challenged by the lack of familiarity and accessibility in the medical community to these powerful tools. To this end, user-friendly automated ML approaches that can facilitate such studies for end-users without extensive data-science training are essential to enable full implementation and widespread use of machine learning capabilities in healthcare. We recently demonstrated the power of such an approach to predicting acute kidney injury and sepsis from complex real-world clinical data using our automated ML platform (MILO: Machine Intelligence Learning Optimizer, Figs. 1 and 2)^21,22^. Here we extend this approach to identify optimized ML models for active TB diagnosis utilizing multi-featured immunologic data*.*

In contrast to the MILO approach, traditional non-automated ML development is time-intensive, requires programming expertise, and relies on human operators to fit a given dataset to a predetermined algorithm which may be less efficient and susceptible to selection bias. MILO eliminates these limitations and improves the accessibility and feasibility of ML-based data science, but more importantly helps identify the optimal ML models while reducing the bias in the process within a transparent platform in each step^23^. Importantly, no a priori assumptions are made using MILO and no programming/ML expertise is required for the operation of the software. Ultimately, MILO uses a combination of unsupervised and supervised machine learning platforms from a large set of algorithms and feature selectors/transformers to create > 1000 unique pipelines (set of automated machine learning steps) yielding > 300,000 models that are then statistically assessed to identify the best performing ML model for a given task. This allows generation of the most suitable ML model (from a range of empirically tested feature set and algorithm combinations) from each unique dataset, rather than attempting to a narrowly predetermined and possibly suboptimal algorithm and feature set. Notably, due to its automated user-interface MILO is accessible to even those users with minimal data science backgrounds. This enables the end-user with familiarity in a given field to select models which best fit their desired application, empowering them to utilize these powerful ML algorithms. This will also enable the broad adoption of such methods across a wide range of scientific disciplines.

## Materials and methods

### Retrospective study population

The retrospective dataset consisted of 199 active pulmonary TB cases (median age 26 years; IQR: 20, 40) reported previously^3^. This dataset was derived from a study conducted to evaluate a multiplex serodiagnostic panel for active tuberculosis in the field in Pakistan. Blood and sputum samples from patients, and healthy individuals (blood samples only) were obtained under the protocols approved through the Institutional Review Boards (IRB) at the School of Biological Sciences (SBS), University of Punjab, Lahore^3^. A written informed consent was obtained from each participant^3^. Plasma samples from patients with active pulmonary TB were collected as previously described^3^. All the patients were from the Gulab Devi Chest Hospital (Lahore, Pakistan) (http://gulabdevihospital.org/), treated following WHO guidelines, administered through the National TB Program (http://www.ntp.gov.pk). All subsequent experiments in this study to analyze tuberculosis related disease biomarkers (blood-based) were performed in accordance with relevant regulations and guidelines approved by the Institutional Review Boards (IRB) at the School of Biological Sciences (SBS), University of Punjab, Lahore^3^. The patients were diagnosed with active pulmonary TB based on a positive result for sputum smear AFB-microscopy (Ziehl–Neelsen Technique), chest X-ray suggestive of TB, and physicians’ assessment based on clinical presentation including persistent cough for more than 2–3 weeks as well as other systemic symptoms when present (fever (low grade and intermittent), weight loss, night sweats, etc.). In addition to sputum smear microscopy, solid culture on LJ (Lowenstein–Jensen) media was performed on sputum samples from all TB patients. Culture is the gold standard which provides bacteriological confirmation. One category of patients included AFB-positive and culture-positive patients (n = 98); and the second category included the AFB-negative and culture-positive patients (n = 101).

Chronic obstructive pulmonary disease (COPD) patients (n = 55) were also included as a control group (in Dataset C only)^3^. The healthy group (n = 79) was comprised of individuals between 21 to 30 years of age, with no history of active TB and no known medical conditions (infection, cancer, or metabolic disease)^3^. They were all from the same geographical area as the TB patients.

### Microbead coating with *M.tb.* antigens

Carboxylated microbeads were purchased from Luminex Corp. (Austin, TX). Various antigen preparations were covalently conjugated to the microbeads as previously described^4,5^. Briefly, an aliquot of 2.5 × 10^6^ beads was removed and resuspended in 80 μl of activation buffer (100 mM monobasic sodium phosphate; pH 6.2) by vortexing and sonication. To activate the beads for cross-linking to proteins, 10 μl of 50-mg/ml sulfo-*N*-hydroxysulfosuccinamide (Pierce, Rockford, IL) and 1-ethyl-3-[3-dimethylaminopropylcarbodiimide (EDC; Pierce, Rockford, IL). The bead mixture was shaken on a rotary shaker at room temperature for 20 min and washed twice with 250 μl phosphate-buffered saline (PBS), pH 7.4. The beads were resuspended in the relevant antigen preparation diluted in PBS buffer and incubated by mixing on a rotator for 2 h at room temperature. Beads were washed twice with 250 μl PBS, resuspended in 250 μl of blocking buffer (1% BSA; 0.1% Tween 20 in PBS, pH 7.4; 0.05% sodium azide), and mixed on a rotator at room temperature for 30 min. After blocking, beads were resuspended in 1 ml of blocking buffer and stored at 2–8 °C in dark. The optimal concentration for each antigen was determined by coupling different microbead sets with 6.25 μg/ml and 25 μg/ml for each HCoV antigen. Bead sets were also coated with bovine serum albumin (BSA, 100 μg/ml) as a negative control protein (Pierce, Rockford, IL) and goat anti-Human IgG (20 μg/ml) as a positive control (Bethyl, TX).

### Multiplex antibody assay

As previously described, recombinant antigens from 28 *M.tb.* genes were expressed in *Escherichia coli*^4,5^. In brief, a mixture of microbead sets, one for each of the coated antigens described below, were incubated with the participants’ plasma specimens, which were diluted 1:200 in 2% Prionex (bio-WORLD, Dublin, OH) for 1 h at room temperature in a 96-well plate. After incubation, the beads were washed twice by adding 100 μl of wash buffer (PBS-tween) per well and drained under vacuum using a vacuum manifold (Millipore Corporation, Bedford, MA). For detection of human IgG, phycoerythrin-conjugated anti-human IgG was used (Jackson ImmunoResearch, Pennsylvania) at a 1:500 dilution in PBS-tween, and incubated at room temperature for 15 min. Following incubation, beads were washed two times with wash buffer, resuspended in 100 μl of wash buffer per well, and subsequently analyzed utilizing the Magpix instrument. This multiplex microbead immunoassay (MMIA) was based on the xMAP technology platform (Luminex Corp, Austin, TX) and was designed to detect the plasma antibodies against each of the targeted *M.tb.* antigens (Rv3881c, Rv0934 (P38), Rv2031c (HspX), Rv1860 (MPT32), Rv3804c (antigen 85a [Ag85a]), Rv1886c (Ag85b), Rv0129c (Ag85c), Rv3875 (ESAT6), Rv3874 (CFP10), Rv3841 (Bfrb1), Rv3418c (GroES), Rv2875 (MPT70), Rv1984c (CFP21), Rv1980c (MPT64), Rv0054, Rv3874-Rv3875 (CFP10-ESAT) fusion, Rv3873, Rv3619, Rv2220, Rv0831c, Rv1009, Rv1099, and Rv2032, Rv1926c, Rv2878c, Rv1677, Rv1566c, Rv3507). Additionally, membrane extracts (MEM) from H37RV, HN879, and CDC1551 *M.tb.* Strains (TB Resource Center at Colorado State University (Fort Collins, CO)) were included in the multiplex panel as well for a total of 31 antigens. The assay was performed as previously detailed; briefly, microbead sets were conjugated to *M.tb.* antigens and multiplex assays were performed^4,5,24^.

### Antibody data

**Figure 3:**
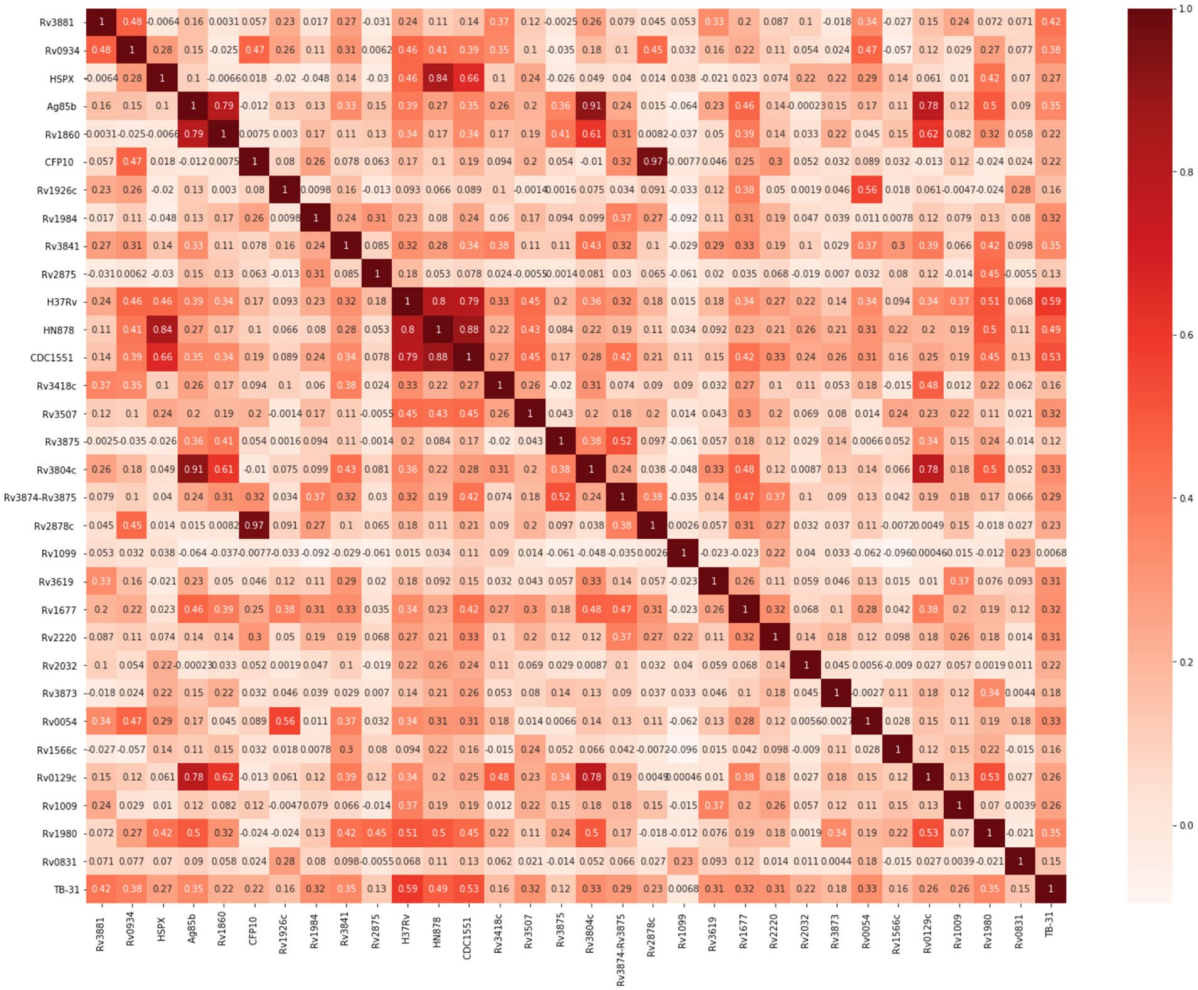
Pearson standard correlation coefficient for TB multiplex microbead immunoassay data. Correlations between the various antigens tested as well as the clinical presence of tuberculosis (here denoted as TB31) is depicted in a heatmap.

Data from a previously published field validation study were collected as median fluorescence intensities (MFI)^3^. These data consist of MFI for all 31 antibodies to known antigens collected in duplicate for 333 plasma samples (TB patients (all culture-positive) n = 199, Healthy n = 79, COPD n = 55) resulting in a total of 20,646 data points. The Pearson standard correlation coefficients between these antigens as well as clinical TB positivity (TB31) are shown in Fig. 3.

### Study design for data analysis using the automated machine learning platform, MILO (machine intelligence learning optimizer)

Analysis of the data was done using Machine Intelligence Learning Optimizer (MILO), an automated machine learning software, an intellectual property of the University of California^16,17^. Figure 1 depicts the MILO platform pipeline for data processing and feature extraction as well as model generation, validation, testing and deployment. For this TB study, the data was split into three datasets as detailed in Table 1. Dataset A (TB n = 62 and Healthy n = 62) was used for the training and initial validation testing in which MILO used a randomly selected subset (80%) of Dataset A for training the model and the remaining 20% for its initial validation, repeated ten times to achieve a 10× k-fold cross validation. The models built on Dataset A were then tested independently on Dataset B (TB n = 137, Healthy n = 17) and Dataset C (TB n = 137, COPD n = 55) to assess each model’s true generalizability on populations similar to the training set (Dataset B), as well as a tertiary dataset (Dataset C) which tested the models on COPD population, not present in the training dataset A. Notably, in this scheme models are never trained on the generalization test set, therefore the results from this validation step are a robust indicator of the classifiers’ true individual performances and less prone to overfitting. This is in contrast to commonly used cross-validation data mining approaches which solely partition the data into training and test sets and ultimately allow training on the whole dataset which can increase the number of overfitted models.

**Table 1:** MILO training/initial validation dataset and generalization datasets.

Dataset name	Dataset type	Dataset composition
Dataset A	Training/validation	Total n = 124 TB n = 62; Healthy n = 62
Dataset B	Generalization/performance evaluation	Total n = 154 TB n = 137; Healthy n = 17
Dataset C^a^	Generalization/performance evaluation^a^	Total n = 192 TB n = 137; COPD n = 55

Training was conducted on Dataset A only (TB and Healthy patients), no COPD cases were used in training. The TB cases (n = 137) are shared between Datasets B and C for validation, however these cases are distinct from the cases in Dataset A (n = 62).

^a^The models were trained on TB and Healthy patient data only. No COPD cases were used in the training phase of these models.

Figure 1 demonstrates the MILO infrastructure which produces an optimized binary classifier and includes an automated data processor (scalers, etc.), a data feature selector (e.g., ANOVA F select percentile feature selector) and data transformer (e.g., principal component analysis), followed by its custom supervised Machine Learning (ML) model builder which includes custom hyperparameter search tools (Grid search along with multiple random search tuners) that help find the best hyperparameter combinations for each of the algorithms within MILO: deep neural network (DNN), logistic regression (LR), Naïve Bayes (NB), k-nearest neighbors (k-NN), support vector machine (SVM), random forest (RF), and XGBoost gradient boosting machine (GBM)^16^. These are followed by automated performance assessment and visualization tools. Ultimately, MILO helps identify the most suitable binary classification ML model(s) from user-defined datasets by simultaneously building a large number of models (> 300,000) through a large set of pipelines (> 1000) which are comprised of various combinations of scalers, scorers, feature selectors and algorithms, ultimately enabling the evaluation of many algorithms and feature combinations for a given dataset. Identification of the final best model using the MILO platform is enabled by the software’s ability to intrinsically calculate the sensitivity, specificity, accuracy, F1 and other predictive values (PPV, NPV) of each of the models from its various individual pipelines based on each model’s performance on the secondary dataset alone (regardless on how well the models were performing in the initial validation test set). The secondary dataset typically represents unbalanced data and may represent the prevalence of the disease (although the unbalanced test dataset B and C used in this pilot study do not represent the true prevalence due to the limited number of healthy subjects available for evaluation). The data in the secondary and tertiary datasets (dataset B and C) was not used in any aspect of the training or model building steps and therefore could be a better measure of the model’s generalizability to real-world applications, limiting the risk of over-fitting. By default, MILO initially highlights the model with the highest average sensitivity and specificity with the highest sensitivity by default upon completion of its analysis step in its results page. However, since all other models’ performance measures are also present, the user can subsequently choose which of all the models is optimal for the purposes of their study (i.e. best ROC-AUC, best F1, etc.). Employing the user-friendly interface of this software (depicted in Fig. 2), they can easily find the particular model that best serves the desired application and clinical need (e.g. the model with best F1 versus a model with best ROC-AUC, etc.). Also, MILO’s transparent platform will display each model’s hyperparameter details which ultimately enables these models to be reproduced in other platforms (e.g. Jupyter Notebook environment) if needed which highlights the transparency of the MILO platform.

Figure 2 highlights the ease-of-use of the MILO graphical interface which allows customizable utilization of core features by users to project specifications (Table 2): the pipelines generated in our automated approach uses seven of the most widely validated and used algorithms (DNN, LR, KNN, SVM, GBM, NB, and RF). The pipelines also include several hyperparameter tuning/search tools including random search tool × 2 in addition to our custom grid search. Notably, random × 2 hyperparameter search tools can outperform most hyperparameter search approaches including grid search and certain Bayesian optimization tools^25^.

**Table 2:** Core features of MILO classification algorithms.

	Automated ML (MILO) approach
Algorithms	KNN, LR, SVM, DNN, RF, NB and GBM
Scaler(s) used	Standard scaler, min/max, and no scaler
Feature selector and/or transformers used	ANOVA F value select percentile (25% increments)
	Random Forest Feature Importances (25% increments) and
	Principal component analysis
Hyperparameter searchers	Grid search and
	Random Search × 2
Scorer(s) used in the training/initial validation phase	Accuracy
	ROC-AUC
	F1
Model assessments	Generalization assessment on all pipelines

Seven of the most widely validated and adopted algorithms (deep neural network (DNN), logistic regression (LR), Naïve Bayes (NB), k-nearest neighbors (k-NN), support vector machine (SVM), random forest (RF), and XGBoost gradient boosting machine (GBM)) are used in the pipelines generated in MILO’s automated approach, which also includes several hyperparameter tuning/search tools such as random search tool × 2 in addition to our custom grid search.

### Application of MILO to training dataset

We defined a “training and initial validation set” and initially built models using MFI values from 124 cases (Dataset A: TB n = 62, healthy n = 62). Features were initially restricted to 11 antigens based on the previous published study (Rv3881, Rv0934, Rv2031c (HspX), Rv1886c (Ag85b), Rv1860, Rv3874 (CFP10), Rv2875, Rv3841, Rv1926c, MEMH37Rv (H37Rv), and Rv1984), preselected out of the 31 antigen panel based on their prior performance using a traditional non-automated ML approach^3^. Using these 11 antigens, an inclusive strategy was used (all algorithms, feature selectors/transformers, hyperparameters searches, and algorithms) to generate models using MILO. Subsequently, for comparative purposes, a separate set of models was also generated using the entire feature set of 31 antigens in order to identify additional potential unique feature sets distinct from the preselected 11 antigens described above.

### Performance of models on secondary and tertiary test sets (generalization datasets)

The models built using the training dataset A developed above were applied to the out-of-sample testing datasets B and C to evaluate the generalizability performance of the models built and evaluated within MILO (Table 1). Dataset B represented out-of-sample testing on 154 total distinct subjects (TB n = 137, Healthy n = 17), while dataset C represented out-of-sample testing on 192 total subjects (TB n = 137, COPD n = 55), and were used to assess the generalization performance of each model with respect to their ability to distinguish TB from healthy and COPD patients, respectively. Notably, the training/initial validation data (Dataset A) did not include COPD patients, therefore these generalization data sets represent a robust stress test of the models. Models generated on the 31-antigen panel as well as the preselected 11 antigen panel were each tested on both of these datasets. Data classification yielded the following measures for the multiplex serology test: true positive (TP), true negative (TN), false positive (FP), and false negative (FN), and testing efficiency (TE) or accuracy. TP provides the measure of the number of positive events positive for *M.tb.* infection and TN provide the number of negative occurrences predicted correctly under a given classification scheme. FP gives an estimate of negative events that are incorrectly predicted to be positive, while the FN estimated the number of TB patients that were predicted negative but were positive^26^. Subsequently, each model’s sensitivity, specificity, ROC-AUC, PPV and NPV along with their F1 values were calculated. The confidence intervals were calculated using the Clopper-Pearson method.

## Results

### Predictive values of different classification algorithms on the testing dataset

As we wanted to establish a model with the best predictive value using our real-world clinical dataset, we implemented a range of algorithms using the MILO platform (Figs. 1 and 2). Approximately 400,000 models were generated from the balanced training and initial testing dataset (dataset A), widely sampling in parallel a range of hyperparameters within various algorithms and feature subsets to discover an optimal solution for discrimination of active TB infection. To robustly assess performance of models, these models were then tested on two distinct out-of-sample datasets (dataset B and dataset C) to acquire commonly used metrics (sensitivity, specificity, ROC-AUC, PPV, NPV, and accuracy) for their generalizability”.

The true performance of the top models generated using all 31 antigens on a test set of 137 TB cases and 17 healthy cases (dataset B) is presented in the “Supplementary Material”, Table S1. Furthermore, the MILO end-to-end model development pipeline resulted in a model requiring a smaller subset of features comprised of the following 23 antigens: Rv3881, Ag85b, Rv1860, CFP10, Rv1984, Rv3841, Rv2875, H37Rv, HN878, CDC1551, Rv3418c, Rv3507, Rv3875, Rv3804c, Rv3874–Rv3875, Rv2878c, Rv1099, Rv3619, Rv2220, Rv3873, Rv0054, Rv1566c, Rv1980. Notably, the following eight antigens were shared in common between the 11 and 23 antigen feature sets: MEMH37Rv (H37Rv), Rv3881, Rv1886c (Ag85b), Rv3874 (CFP10), Rv1980, Rv1860, Rv1984, Rv3841, Rv2875. Table 3 highlights the Pearson correlation between these two antigen groups and their shared antigens and respective correlation coefficients with respect to patient’s clinical TB status. This logistic regression model afforded superior performance utilizing only 75% of the overall features (i.e. 23 features out of the 31 initial features provided to MILO). This model utilized random forest importance feature selection to highlight a more precise set of 23 antigens (out of 31 selected antigens listed in Table 3) and facilitate optimal classification performance in comparison to the other models as noted by improved sensitivity and specificity (Table 4). The performance of the 23 feature model selected from sampling of all available features was also superior when compared with the best models developed from more limited feature sets representing the top 2, 4, 8, and 16 features based on Pearson correlation (Table 3) as well as manually selected top feature sets (Supplemental Table S3 and Supplemental Fig. 1). Therefore, the feature selection process undertaken by MILO identified a specific feature set which enabled optimal extraction of interpretable information, rather than selecting the maximal number of features with redundant information, or not including low correlation features which may still improve the model.

**Table 3:** Selected antigens for 11 and 23 feature sets for MILO models ranked by pearson correlation coefficient to clinical TB status.

Feature	Correlation to clinical TB Status	Included in 11 feature set	Included in 23 feature set
H37Rv (MEMH37Rv)	0.59	**Y**	**Y**
CDC1551	0.53	N	Y
HN878	0.49	N	Y
Rv3881	0.42	**Y**	**Y**
Rv0934	0.38	Y	N
Ag85b (Rv1886c)	0.35	**Y**	**Y**
Rv3841	0.35	**Y**	**Y**
Rv1980	0.35	N	Y
Rv3804c	0.33	N	Y
Rv0054	0.33	N	Y
Rv1984	0.32	**Y**	**Y**
Rv3507	0.32	N	Y
Rv1677	0.32	N	N
Rv3619	0.31	N	Y
Rv2220	0.31	N	Y
Rv3874-Rv3875	0.29	N	Y
HSPX (Rv2031c)	0.27	Y	N
Rv0129c	0.26	N	N
Rv1009	0.26	N	N
Rv2878c	0.23	N	Y
Rv1860	0.22	**Y**	**Y**
CFP10 (Rv3874)	0.22	**Y**	**Y**
Rv2032	0.22	N	N
Rv3873	0.18	N	Y
Rv1926c	0.16	Y	N
Rv3418c	0.16	N	Y
Rv1566c	0.16	N	Y
Rv0831	0.15	N	N
Rv2875	0.13	**Y**	**Y**
Rv3875	0.12	N	Y
Rv1099	0.0068	N	Y

The bold represents the features that were present in both the 11 feature set and the 23 feature set data.

**Table 4:** Performance comparisons of the 11 preselected antigens vs 23 antigens selected by MILO.

	TB vs healthy subjects ONLY (Dataset B)			TB vs non-TB COPD subjects ONLY (Dataset C)		
	Statistic	Value (%)	95% CI (%)	Statistic	Value (%)	95% CI (%)
TB best MILO model using **11** antigens Found through MILO to be a Neural-network Model	Sensitivity	**83.94**	76.70–89.65	Sensitivity	**83.94**	76.70–89.65
	Specificity	**100.00**	80.49–100.00	Specificity	**76.36**	62.98–86.77
	Disease prevalence	88.96	82.91–93.44	Disease prevalence	71.35	64.40–77.63
	PPV	**100.00**		PPV	**89.84**	84.54–93.47
	NPV	**43.59**	34.51–53.12	NPV	**65.62**	55.89–74.21
	Accuracy	**85.71**	79.17–90.83	Accuracy	**81.77**	75.57–86.96
TB best MILO model using *23* antigens (the best of the 31 antigens) Found through MILO to be a Logistic Regression Model	Sensitivity	*90.51*	84.32–94.85	Sensitivity	*90.51*	84.32–94.85
	Specificity	*100.00*	80.49–100.00	Specificity	*74.55*	61.00–85.33
	Disease prevalence	88.96	82.91–93.44	Disease prevalence	71.35	64.40–77.63
	PPV	*100.00*		PPV	*89.86*	84.89–93.32
	NPV	*56.67*	43.81–68.69	NPV	*75.93*	64.77–84.40
	Accuracy	*91.56*	86.00–95.43	Accuracy	*85.94*	80.20–90.52

The bold values represent the performance measures for the best model using 11 antigens while the italicized values represent the performance measures for the best model using 23 antigens.

**Figure 4:**
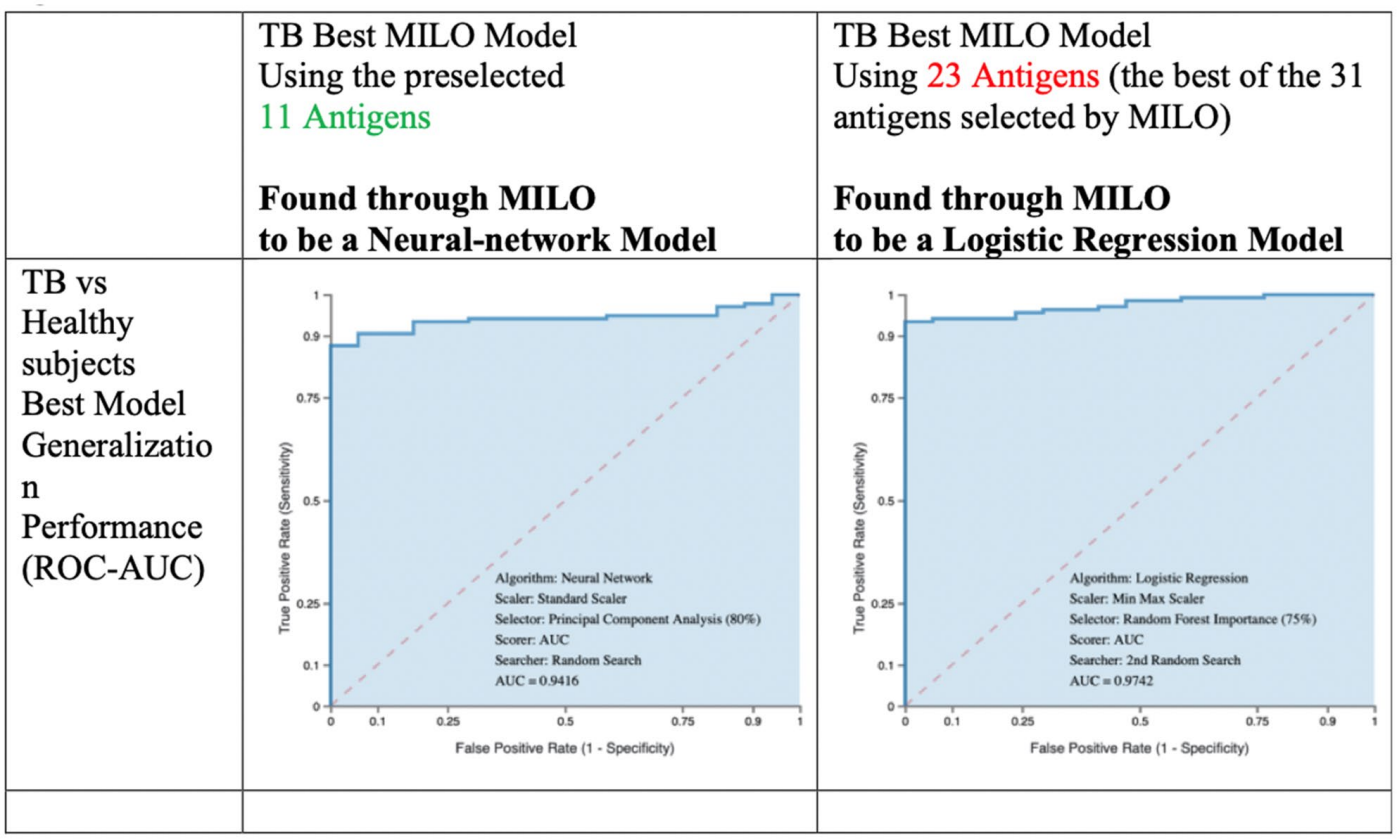
Receiver operator characteristic (ROC) curve for MILO generated models using 11 preselected antigens vs 23 antigens (out of 31-plex). The best model for 11 preselected antigen panel was a neural network model and the best model for 23 antigen panel (found through MILO’s unsupervised arm) was a logistic regression model as shown in the ROC-AUC graphs.

The performance of a model which utilized the subset 23 antigen panel selected by MILO was compared in detail against the model generated by the limited and preselected panel of 11 antigens utilized in a prior study (listed in Supplementary Table S1). Figure 4 shows the receiver operator characteristic (ROC) curve which compares the performance characteristics of the best models for the 23 antigens panel versus the preselected 11 antigen panel (out of the 31 total antigens evaluated). The best model for the 11 preselected antigen panel was a neural network model while a logistic regression model offered the best performance for the 23 antigen panel. The above models were selected from amongst ~ 400,000 total models that were generated by MILO using the seven distinct algorithms (DNN, LR, NB, k-NN, SVM, RF, and XGBoost GBM) that were described above.

The performance comparisons of the models trained and initially validated on dataset A were then secondarily validated on dataset B for generalizability performance testing (to distinguish TB from healthy subjects). Following this, models trained and initially validated on dataset A were also validated on tertiary dataset C to assess their performance on previously unseen COPD patients as shown in Table 4*.*

Using the preselected 11 *M.tb.* antigens, the best performing model’s (based on a neural network) sensitivity and specificity on the out-of-sample dataset B test data was 84% and 100% respectively (Table 4). In contrast, this model showed a sensitivity and specificity of 84% and 76% respectively on the out-of-sample dataset C test data (distinguishing TB from non-TB COPD patients). Results for datasets with the 11 preselected antigens on healthy (dataset B) and COPD containing controls (dataset C) were 100% and 90% for PPV, 44% and 66% for NPV, and 86% and 82% for accuracy, respectively.

Using the same study approach as described above MILO was also able to find an optimized model which utilized only 75% of the total features (23 out of the 31 antigens). In contrast to the best performing model trained using the 11 antigens feature set (derived from a neural network algorithm), the best performing model on the 23 feature set was based on a logistic regression algorithm (Table 4). This 23-antigen model showed superior performance over the 11 preselected antigen model for sensitivity, PPV, NPV, and accuracy. Testing of the 23 antigen model on dataset B yielded a sensitivity of 91% (7% higher than the 11 antigen model) with a specificity of 100%. The NPV was 57% (13% higher) with an identical PPV of 100% and an accuracy of 92% (6% higher). When tested on the COPD-included dataset C, the 23 antigen model demonstrated a sensitivity of 91% (7% higher than 11 feature model), a specificity of 75% (1% less than 11 feature model), a NPV of 76% (10% higher than the NPV), a similar PPV (90%), and an accuracy of 86% (4% higher).

## Discussion

Modern methods in data mining have proven to be useful for comparison of the prediction power of different models derived from a range of feature selectors, searchers, and algorithm types. Machine learning techniques provide powerful methods which enable full extraction of information from complex datasets. The empiric models that are generated from such approaches provide a robust, reproducible, and cost-effective means for analyzing the multiplexed immunologic data generated from high-throughput MMIA assays. These validated models can improve sensitivity and specificity for TB serological assays, enhancing existing experimental approaches through optimal application of data science methods. Ultimately, the synergistic combination of these powerful experimental methods and predictive ML models can provide reliable and actionable interpretation of patient active TB status for physicians in high-burden TB countries.

In this study, we generated models by using an automated machine learning platform, MILO, to assess large quantities of data generated in the multiplex immunoassay system. We defined a training and initial validation test set (dataset A) based on the validated retrospective experimental data (simultaneous detection of antibodies to 31 antigens) from 124 subjects (TB patients n = 62, Healthy n = 62)^3^. The secondary and tertiary testing datasets consisted of dataset B and dataset C which was comprised of 209 subjects overall (TB n = 137, Healthy n = 17, COPD n = 55). A cumulative validation of these results on this pilot study using MILO allowed efficient quality control and assessment of the model’s true performance measures and generalizability.

As expected, increasing the number of features used by MILO for model selection generally improved AUC (Fig. 4) and surprisingly the best model was able to yield these results utilizing only a subset of features (75%, 23 of 31 total). The 23 antigen MILO model showed superior performance over the 11 preselected antigen MILO model for sensitivity, PPV, NPV, and accuracy (Table 3). For the 23-antigen model, sensitivity for TB patients was 91% which is 7% higher than the 11 antigen model (Table 4). However, as noted above, the top model utilized only 75% of the total features, indicating that MILO was able to efficiently extract specific determinants of infection rather than being dependent on all available data. This is critical since clinical implementation of diagnostics ideally will use only the necessary number of antigens to conserve resources.

Sensitivity using the 23 antigen MILO model (Table 4) was similar (91% in each case) compared to a previously developed 11 antigen traditional modified Decision Tree algorithm. Specificity for the 23 antigen MILO model was superior (100% vs 91% respectively) for TB versus healthy subjects but not surprisingly resulted in lower specificity, PPV, and NPV for TB vs Non-TB COPD subjects since the models were not trained on COPD patients (75% vs 96%, 90% vs 96%, and 76% vs 86%, respectively). Notably, the prior study used a reduced three-fold validation of data and model performance was not assessed on out-of-sample data (the data set aside in this study as datasets B and C), raising the possibility of over-fitting which was dealt with here with our two additional out-of-sample datasets (datasets B and C). Additionally, the prior model that was published on the 11 preselected antigens was directly trained on COPD data^3^, whereas MILO as previously noted was tested, but not trained on this COPD data. Therefore, the performance of the MILO-generated model on COPD data is impressive given the disadvantage posed by not training on any COPD patients. This increases confidence that the determinants of TB infection utilized by this model are robust.

Our approach demonstrates similar performance from testing on out-of-sample and generalization datasets, which provides a robust stress test of the model reflecting real-world performance with minimal risk of overfitting. To test the effect of the model performance based on the same out of sample datasets (dataset B and dataset C) used in our 23 feature set models, we also used MILO to find the best performing model based on the predetermined 11 features noted above from the previously published study. This approach enabled us to objectively compare the two groups (the 23 feature set versus the 11 predetermined feature set) within MILO (Table 4). This approach displayed that the sensitivity of the best model based on the 11 features (tested on out-of-sample data from dataset B and C) was lower than the 23 feature set found by MILO’s approach (84% versus 91%). Notably, the specificities of these models were now found to be nearly identical when using performance measures that were based on the out-of-sample data with both 11 and 23 features showing 100% specificities and PPVs when tested on dataset B (TB vs. Healthy) while the performance on dataset C (TB vs COPD) showed similar respective specificities of 7–6% and 75% and PPVs of 90%. Importantly, this approach also highlighted the improved NPV of the 23 feature set model as compared to the best model identified through MILO based on the predetermined 11 feature set model (57% versus 44% when (Table 4).

## Limitations of our study

In this study, models were not trained on the COPD data due to the modest sample size of this cohort. A follow-up study with additional COPD samples would enable training and testing on datasets with TB and COPD patients which would likely improve model performance in this more difficult patient population (while hopefully retaining performance in the healthy patient population). Since discrimination between TB and COPD patients may more accurately reflect the clinical context in which these models would be deployed, additional studies are needed to further develop optimized computational models for this application. Additionally, larger datasets may provide more robust extraction of subtle patterns among feature sets which may allow training of models which utilize even fewer features, enabling easier clinical deployment.

Interestingly, of the 23 antigen features used by MILO in the top model, only eight antigens overlap with the set of 11 selected antigen features from the previously published algorithm. This implies that rather than improving model performance by simply adding additional features, diverse models developed from a range of parent algorithms, feature classifiers, and parameter searchers rely on orthogonal intrinsic characteristics of the dataset to yield classification results. Additionally, it must be noted that some of the selected features include those with relatively low individual correlation to TB status (Table 3). This again suggests that ML approaches enhance interpretation of complex data sets by extracting information from data which would appear to be dispensable (features with minimal correlation to TB status by traditional statistical measures). Therefore, widespread sampling of models through empiric methods has the inherent advantage of allowing discovery of various patterns within a dataset, since a priori the optimal combination of parameters, features, searchers, and algorithms cannot be known. Selection from among a large set of models based on empiric performance metrics allows individualized optimization of the computation classifiers for a given application (maximizing sensitivity or specificity). Clinical interpretation of high-volume, complex, multi-featured datasets requires rapid and objective identification of specific disease-associated patterns by reproducible classifiers. Although this task can be accomplished by traditional data science methods guided by experienced practitioners, the optimal application of machine learning (ML) methods to multi-dimensional data generates classifiers with improved diagnostic specificity and sensitivity as demonstrated here by the application of MILO to MMIA datasets in our TB patients. This approach reveals serological patterns otherwise obscured by the sheer enormity and complexity of large data volumes. Indeed, without optimal downstream interpretation, the diagnostic value of complex datasets may be underutilized. The combination of high-throughput acquisition of quantitative multidimensional serologic data with robust ML-derived classifiers provides a powerful approach to timely, accurate, and reproducible clinical diagnosis of TB as needed for appropriate treatment of this critical public health concern, particularly in endemic resource-poor areas.

The rapid (timeframe of hours to days), empiric, and automated nature of the MILO platform has several advantages over traditional non-automated data science methods. First, the speed of this method enables near-immediate analysis of complex datasets as required in particular in the setting of emerging infectious diseases or pandemics. Whether the causative agent is a wholly unknown virus (such as COVID-19) or a mutated variant of a previously characterized agent (seasonal influenza), timely generation and deployment of robust ML-built models represent an invaluable tool for researchers in data analysis and clinical interpretation. Additionally, the inherently automated and user-friendly nature of the MILO platform makes ML methods accessible to end-users who may select from among hundreds of thousands of models based on the desired characteristics of the classifier (maximizing either sensitivity, specificity, F1, or ROC-AUC) without the need for time-intensive manual application of traditional methods requiring significant data science expertise. Also, since it is expected that some infectious agents evolve rapidly, the fast turnaround time of this approach allows for expeditious generation, validation, and deployment of new models. Although there are significant advantages in efficiency, performance, and accessibility offered by the MILO platform, there are operational parameters which do limit its appropriate utilization. Input data is restricted to numerical datasets, (the platform does not support image-based analysis), and performs binary classification only, therefore multi-class problems are not supported by this approach currently. No imputation function is available, therefore after dataset importation missing values will result in smaller datasets for development. Therefore, although MILO represents an end-to-end solution, ensuring data quality and completeness prior to input is critical. Additionally, although MILO is able to discover optimal feature sets across a variety of algorithms empirically through generous sampling, this approach is computationally intensive, therefore this architecture is limited to a training dataset size of 20,000 cases to ensure run completion within an adequate period of time. This will not typically represent an issue for the smaller clinical datasets for which MILO was designed, however for larger datasets this may not be optimal.

This study focused on the application of an automated ML method (MILO) to multiplex *M. tb* serologic data, successfully generating viable and robust classifiers to provide actionable clinical interpretation of active TB infection. This proof of concept study represents a significant step towards improving diagnostic capabilities as required in this critical and long-standing global health struggle and justifies continued development of optimized classifiers using larger COPD-included datasets. Moreover, this work supports the broad application of our automated ML platform (MILO) to computational analysis of large volumes of data. Although such data is commonly produced from a variety clinical translational research disciplines (proteomics, metabolomics, genomics), here we demonstrate that these methods may be particularly suited for applications in infectious disease due to the time-sensitive nature of analysis and interpretation of data as demanded by public health needs.

## Supplementary Information

Supplementary Information.

## Data Availability

The data used in this study has previously been made available as a Supplemental file (S1 Appendix) from the following publication (Khaliq et al.^3^). The data is also available from the authors upon reasonable request.
