# Supplementary material for: Automated machine learning for endemic active tuberculosis prediction from multiplex serological data
Source: Sci Rep. 2021 Sep 9;11:17900. doi: 10.1038/s41598-021-97453-7 (PMC8429671; doi:10.1038/s41598-021-97453-7)

**Automated Machine Learning for Endemic Active Tuberculosis Prediction from Multiplex Serological Data**

**Hooman H. Rashidi^1^*, Luke T. Dang^1^, Samer Albahra^1^, Resmi Ravindran^1^ & Imran H. Khan^1^***

**Author Affiliations:**

**1) Department of Pathology and Laboratory Medicine, University of California, Davis, 4400 V Street, Sacramento, CA, 95817, USA.**

***Corresponding authors: Hooman H. Rashidi** [**hrashidi@ucdavis.edu**](mailto:hrashidi@ucdavis.edu) **& Imran Khan** [**ihkhan@ucdavis.edu**](mailto:ihkhan@ucdavis.edu)

**Supplemental Data:**

**Supplementary Table S1: Performance evaluations of the test set (dataset B) using the training set from the 31 antigens initially evaluated for TB and Healthy**

| **Algorithm** | **Feature selector** | **Searcher** | **Scorer** | **Sensitivity** | **Specificity** | **# of Antigens** | **Selected Antigens** |
| --- | --- | --- | --- | --- | --- | --- | --- |
| logistic regression | random forest importance (75%) | 2nd random search | AUC | 0.9051 | 1 | 23 | ['Rv3881', 'Ag85b', 'Rv1860', 'CFP10', 'Rv1984', 'Rv3841', 'Rv2875', 'H37Rv', 'HN878', 'CDC1551', 'Rv3418c', 'Rv3507', 'Rv3875', 'Rv3804c', 'Rv3874-Rv3875', 'Rv2878c', 'Rv1099', 'Rv3619', 'Rv2220', 'Rv3873', 'Rv0054', 'Rv1566c', 'Rv1980'] |
| random forest | principal component analysis (90%) | grid search | accuracy | 0.9051 | 0.9412 | 31 | ['Rv3881', 'Rv0934', 'HSPX', 'Ag85b', 'Rv1860', 'CFP10', 'Rv1926c', 'Rv1984', 'Rv3841', 'Rv2875', 'H37Rv', 'HN878', 'CDC1551', 'Rv3418c', 'Rv3507', 'Rv3875', 'Rv3804c', 'Rv3874-Rv3875', 'Rv2878c', 'Rv1099', 'Rv3619', 'Rv1677', 'Rv2220', 'Rv2032', 'Rv3873', 'Rv0054', 'Rv1566c', 'Rv0129c', 'Rv1009', 'Rv1980', 'Rv0831'] |
| gradient boosting machine | principal component analysis (90%) | grid search | accuracy | 0.8905 | 1 | 31 | ['Rv3881', 'Rv0934', 'HSPX', 'Ag85b', 'Rv1860', 'CFP10', 'Rv1926c', 'Rv1984', 'Rv3841', 'Rv2875', 'H37Rv', 'HN878', 'CDC1551', 'Rv3418c', 'Rv3507', 'Rv3875', 'Rv3804c', 'Rv3874-Rv3875', 'Rv2878c', 'Rv1099', 'Rv3619', 'Rv1677', 'Rv2220', 'Rv2032', 'Rv3873', 'Rv0054', 'Rv1566c', 'Rv0129c', 'Rv1009', 'Rv1980', 'Rv0831'] |
| gradient boosting machine | principal component analysis (90%) | grid search | F1 | 0.8905 | 1 | 31 | ['Rv3881', 'Rv0934', 'HSPX', 'Ag85b', 'Rv1860', 'CFP10', 'Rv1926c', 'Rv1984', 'Rv3841', 'Rv2875', 'H37Rv', 'HN878', 'CDC1551', 'Rv3418c', 'Rv3507', 'Rv3875', 'Rv3804c', 'Rv3874-Rv3875', 'Rv2878c', 'Rv1099', 'Rv3619', 'Rv1677', 'Rv2220', 'Rv2032', 'Rv3873', 'Rv0054', 'Rv1566c', 'Rv0129c', 'Rv1009', 'Rv1980', 'Rv0831'] |
| gradient boosting machine | principal component analysis (80%) | grid search | accuracy | 0.8905 | 0.9412 | 31 | ['Rv3881', 'Rv0934', 'HSPX', 'Ag85b', 'Rv1860', 'CFP10', 'Rv1926c', 'Rv1984', 'Rv3841', 'Rv2875', 'H37Rv', 'HN878', 'CDC1551', 'Rv3418c', 'Rv3507', 'Rv3875', 'Rv3804c', 'Rv3874-Rv3875', 'Rv2878c', 'Rv1099', 'Rv3619', 'Rv1677', 'Rv2220', 'Rv2032', 'Rv3873', 'Rv0054', 'Rv1566c', 'Rv0129c', 'Rv1009', 'Rv1980', 'Rv0831'] |
| gradient boosting machine | principal component analysis (90%) | grid search | accuracy | 0.8905 | 0.9412 | 31 | ['Rv3881', 'Rv0934', 'HSPX', 'Ag85b', 'Rv1860', 'CFP10', 'Rv1926c', 'Rv1984', 'Rv3841', 'Rv2875', 'H37Rv', 'HN878', 'CDC1551', 'Rv3418c', 'Rv3507', 'Rv3875', 'Rv3804c', 'Rv3874-Rv3875', 'Rv2878c', 'Rv1099', 'Rv3619', 'Rv1677', 'Rv2220', 'Rv2032', 'Rv3873', 'Rv0054', 'Rv1566c', 'Rv0129c', 'Rv1009', 'Rv1980', 'Rv0831'] |
| gradient boosting machine | principal component analysis (90%) | grid search | F1 | 0.8905 | 0.9412 | 31 | ['Rv3881', 'Rv0934', 'HSPX', 'Ag85b', 'Rv1860', 'CFP10', 'Rv1926c', 'Rv1984', 'Rv3841', 'Rv2875', 'H37Rv', 'HN878', 'CDC1551', 'Rv3418c', 'Rv3507', 'Rv3875', 'Rv3804c', 'Rv3874-Rv3875', 'Rv2878c', 'Rv1099', 'Rv3619', 'Rv1677', 'Rv2220', 'Rv2032', 'Rv3873', 'Rv0054', 'Rv1566c', 'Rv0129c', 'Rv1009', 'Rv1980', 'Rv0831'] |
| gradient boosting machine | principal component analysis (90%) | 2nd random search | F1 | 0.8905 | 0.9412 | 31 | ['Rv3881', 'Rv0934', 'HSPX', 'Ag85b', 'Rv1860', 'CFP10', 'Rv1926c', 'Rv1984', 'Rv3841', 'Rv2875', 'H37Rv', 'HN878', 'CDC1551', 'Rv3418c', 'Rv3507', 'Rv3875', 'Rv3804c', 'Rv3874-Rv3875', 'Rv2878c', 'Rv1099', 'Rv3619', 'Rv1677', 'Rv2220', 'Rv2032', 'Rv3873', 'Rv0054', 'Rv1566c', 'Rv0129c', 'Rv1009', 'Rv1980', 'Rv0831'] |
| random forest | principal component analysis (80%) | grid search | accuracy | 0.8905 | 0.9412 | 31 | ['Rv3881', 'Rv0934', 'HSPX', 'Ag85b', 'Rv1860', 'CFP10', 'Rv1926c', 'Rv1984', 'Rv3841', 'Rv2875', 'H37Rv', 'HN878', 'CDC1551', 'Rv3418c', 'Rv3507', 'Rv3875', 'Rv3804c', 'Rv3874-Rv3875', 'Rv2878c', 'Rv1099', 'Rv3619', 'Rv1677', 'Rv2220', 'Rv2032', 'Rv3873', 'Rv0054', 'Rv1566c', 'Rv0129c', 'Rv1009', 'Rv1980', 'Rv0831'] |
| random forest | principal component analysis (80%) | grid search | AUC | 0.8905 | 0.9412 | 31 | ['Rv3881', 'Rv0934', 'HSPX', 'Ag85b', 'Rv1860', 'CFP10', 'Rv1926c', 'Rv1984', 'Rv3841', 'Rv2875', 'H37Rv', 'HN878', 'CDC1551', 'Rv3418c', 'Rv3507', 'Rv3875', 'Rv3804c', 'Rv3874-Rv3875', 'Rv2878c', 'Rv1099', 'Rv3619', 'Rv1677', 'Rv2220', 'Rv2032', 'Rv3873', 'Rv0054', 'Rv1566c', 'Rv0129c', 'Rv1009', 'Rv1980', 'Rv0831'] |
| random forest | principal component analysis (80%) | grid search | F1 | 0.8905 | 0.9412 | 31 | ['Rv3881', 'Rv0934', 'HSPX', 'Ag85b', 'Rv1860', 'CFP10', 'Rv1926c', 'Rv1984', 'Rv3841', 'Rv2875', 'H37Rv', 'HN878', 'CDC1551', 'Rv3418c', 'Rv3507', 'Rv3875', 'Rv3804c', 'Rv3874-Rv3875', 'Rv2878c', 'Rv1099', 'Rv3619', 'Rv1677', 'Rv2220', 'Rv2032', 'Rv3873', 'Rv0054', 'Rv1566c', 'Rv0129c', 'Rv1009', 'Rv1980', 'Rv0831'] |
| random forest | principal component analysis (80%) | random search | accuracy | 0.8905 | 0.9412 | 31 | ['Rv3881', 'Rv0934', 'HSPX', 'Ag85b', 'Rv1860', 'CFP10', 'Rv1926c', 'Rv1984', 'Rv3841', 'Rv2875', 'H37Rv', 'HN878', 'CDC1551', 'Rv3418c', 'Rv3507', 'Rv3875', 'Rv3804c', 'Rv3874-Rv3875', 'Rv2878c', 'Rv1099', 'Rv3619', 'Rv1677', 'Rv2220', 'Rv2032', 'Rv3873', 'Rv0054', 'Rv1566c', 'Rv0129c', 'Rv1009', 'Rv1980', 'Rv0831'] |
| random forest | principal component analysis (80%) | random search | F1 | 0.8905 | 0.9412 | 31 | ['Rv3881', 'Rv0934', 'HSPX', 'Ag85b', 'Rv1860', 'CFP10', 'Rv1926c', 'Rv1984', 'Rv3841', 'Rv2875', 'H37Rv', 'HN878', 'CDC1551', 'Rv3418c', 'Rv3507', 'Rv3875', 'Rv3804c', 'Rv3874-Rv3875', 'Rv2878c', 'Rv1099', 'Rv3619', 'Rv1677', 'Rv2220', 'Rv2032', 'Rv3873', 'Rv0054', 'Rv1566c', 'Rv0129c', 'Rv1009', 'Rv1980', 'Rv0831'] |

**Supplemental Table S2. Comparison of our Non-automated versus Automated machine learning Approach**

**The table highlights the similarities and differences between the two machine-learning approaches (the traditional non-automated approaches versus our automated MILO approach). * KNN, LR, SVM, DNN, RF, NB and GBM (K- nearest neighbors, logistic regression, support vector machine, random forest, naïve Bayes, and gradient boosting machine).**

|  | Non-Automated ML Approach | Our Automated ML (MILO) Approach |
| --- | --- | --- |
| Algorithms* | **One or more of the following algorithms (resource-dependent) : KNN, LR, SVM, NN, NB, GBM and RF** | **All of the following algorithms being evaluated simultaneously: KNN, LR, SVM, DNN, RF, NB and GBM** |
| Scaler(s) used | **Standard scaler (default scaler used by many studies)** | **3 scaling options utilized: standard scaler, Min/max scaler, and no scaler** |
| Feature Selector and/or transformers used | **For smaller datasets, traditional approach may be to utilize all features, or to utilize a single feature selection tool rather than multiple simultaneous models.** | **Multiple Unsupervised ML methods are incorporated within MILO’s supervised ML pipelines using all the algorithms noted above since MILO makes no assumption of which feature sets (i.e. all or subset of the features) are most important for attaining the best performing ML model. The 3 separate Unsupervised ML methods embedded in MILO:**   - **ANOVA F Value Select percentile (25% increments)** - **Random Forest Feature Importances (25% increments) and** - **Principal Component Analysis** |
| Hyperparameter Searchers | **Employs a Grid search or some other single hyperparameter searcher** | **MILO incorporates multiple searchers:**   - **Custom Grid search and** - **Random Search x2 (since studies have shown that hyperparameter searcher employing multiple random search windows will typically outperform many of the single hyperparameter search tools; see reference below)** |
| Scorer(s) used in the training/initial validation phase | **Accuracy (since that is typically the default scorer utilized in the training phase/ initial validation and the cross-validation step)** | **MILO incorporates multiple scorers (making no assumptions above which scorer being best in its training phase/ initial validation and the cross-validation steps). The 3 scorers evaluated simultaneously include:**   - **Accuracy score** - **ROC-AUC score** - **F1 score** |
| Model Assessments | **Generalization assessment is based on one’s limited number of ML pipelines that can be generated (i.e. 10-100) generated (combination of different algorithms, scalers, hyperparameter searchers, feature selectors and scorers) that ultimately give rise to a limited number of optimized models.** | **Generalization assessment on all possible ML pipelines (>1500) generated from the above (combination of different algorithms, scalers, hyperparameter searchers, feature selectors and scorers) that ultimately give rise to thousands of optimized models.** |

**Supplemental Table S3. Comparison of model performance based on number of biomarkers utilized by MILO in model development (best model for each).**

| Metric | Manually Selected Top 2 Features | Manually Selected Top 4  Features | Top 2  Features (r) | Top 4f  Features  (r) | Top 8  Features  (r) | Top 16  Features  (r) | All 31 Features  (r) | Prior Model 11 Features | MILO  Top 23  Features |
| --- | --- | --- | --- | --- | --- | --- | --- | --- | --- |
| Sensitivity | 73 | 73 | 74 | 77 | 77 | 85 | 91 | 84 | 91 |
| Specificity | 100 | 100 | 100 | 100 | 100 | 94 | 94 | 100 | 100 |
| ROC-AUC | 92 | 93 | 91 | 93 | 94 | 94 | 95 | 94 | 97 |
|  |  |  |  |  |  |  |  |  |  |

(r) = Pearson’s Correlation Coefficient value

**Supplemental Figure 1. Comparison of model performance based on number of biomarkers utilized by MILO in model development (best model for each).**


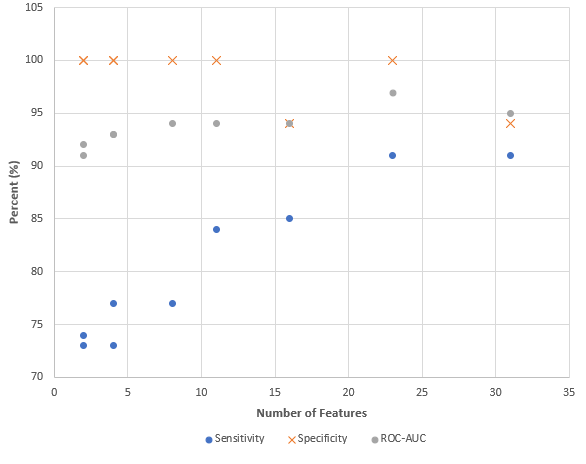

Supplement: Supplementary file 1 — Supplementary Information. [file 41598_2021_97453_MOESM1_ESM.docx]
